# A Review of Needle Navigation Technologies in Minimally Invasive Cardiovascular Surgeries—Toward a More Effective and Easy-to-Apply Process

**DOI:** 10.3390/diagnostics15020197

**Published:** 2025-01-16

**Authors:** Katharina Steeg, Gabriele Anja Krombach, Michael Horst Friebe

**Affiliations:** 1 Department of Diagnostic and Interventional Radiology, University Hospital Giessen, Justus-Liebig-University Giessen, Klinikstraße 33, 35392 Giessen, Germany; 2 Faculty of Computer Science, AGH University Kraków, 30-059 Kraków, Poland; friebe@agh.edu.pl; 3 INKA Innovation Lab, Faculty of Medicine, Otto-von-Guericke-University, 39120 Magdeburg, Germany

**Keywords:** needle navigation technology, needle guidance, multimodal needle guidance, vibroacoustic signal processing

## Abstract

**Background:** This review evaluates needle navigation technologies in minimally invasive cardiovascular surgery (MICS), identifying their strengths and limitations and the requirements for an ideal needle navigation system that features optimal guidance and easy adoption in clinical practice. **Methods:** A systematic search of PubMed, Web of Science, and IEEE databases up until June 2024 identified original studies on needle navigation in MICS. Eligible studies were those published within the past decade and that performed MICS requiring needle navigation technologies in adult patients. Animal studies, case reports, clinical trials, or laboratory experiments were excluded to focus on actively deployed techniques in clinical practice. Extracted data included the study year, modalities used, procedures performed, and the reported strengths and limitations, from which the requirements for an optimal needle navigation system were derived. **Results:** Of 36 eligible articles, 21 used ultrasound (US) for real-time imaging despite depth and needle visibility challenges. Computer tomography (CT)-guided fluoroscopy, cited in 19 articles, enhanced deep structure visualization but involved radiation risks. Magnetic resonance imaging (MRI), though excellent for soft-tissue contrast, was not used due to metallic tool incompatibility. Multimodal techniques, like US–fluoroscopy fusion, improved accuracy but added cost and workflow complexity. No single technology meets all the criteria for an ideal needle navigation system, which should combine real-time imaging, 3D spatial awareness, and tissue integrity feedback while being cost-effective and easily integrated into existing workflows. **Conclusions:** This review derived the criteria and obstacles an ideal needle navigation system must address before its clinical adoption, along with novel technological approaches that show potential to overcome those challenges. For instance, fusion technologies overlay information from multiple visual approaches within a single interface to overcome individual limitations. Additionally, emerging diagnostic methods like vibroacoustic sensing or optical fiber needles offer information from complementary sensory channels, augmenting visual approaches with insights into tissue integrity and structure, thereby paving the way for enhanced needle navigation systems in MICS.

## 1. Introduction

In 2021, 10.8 million deaths were related to cardiovascular diseases (CVDs), representing approximately 31% of all global deaths and a leading cause of mortality worldwide [1]. Treatment methodologies for CVDs include traditional invasive surgeries as well as minimally invasive cardiovascular surgeries (MICSs).

In contrast to traditional invasive surgeries, which involve large openings and often a sternotomy for direct access to the heart or major blood vessels, MICSs are performed through small incisions or catheters, typically only under local anesthesia [2]. The reduced trauma associated with MICS leads to shorter hospital stays, quicker recovery times, and lower overall complication rates [3].

Nevertheless, a challenge of MICS is the accurate navigation of medical instruments through complex anatomical landscapes to the target site while avoiding certain structures and procedural complications [4]. For navigation, imaging technologies such as ultrasound (US) and computed tomography (CT), including fluoroscopy, conventional guided X-ray fluoroscopy, and magnetic resonance imaging (MRI), are commonly used and well-established modalities, with each offering unique advantages and limitations.

US is widely available and relatively inexpensive, offers good soft-tissue contrast and no radiation exposure but has limited visualization of interventional tools, as the field of view varies with the probe’s position [5,6,7]. Fluoroscopy provides real-time X-ray imaging, but just like CT, it exposes patients and doctors to radiation. MRI delivers high-resolution images without radiation exposure but is only rarely available for needle procedures in MICS. It comes with very high costs and requires dedicated support systems and surgical instruments [8].

The chosen visualization technique is crucial for a safe procedure, as is the selection of the access site to the cardiovascular system and the tools used. Currently, no single technology meets all requirements for the accurate navigation and deployment of medical tools during cardiovascular interventions, and significant challenges impede the development, adoption, and utilization of new technologies.

Given the above, this review examines the current state of needle navigation technologies actively deployed in MICS and identifies the technical limitations of existing methods. By discussing the operational challenges related to training and workflow integration and highlighting innovative approaches, this review aims to provide a comprehensive overview of how needle navigation can be improved to provide more cost-efficient solutions and better serve healthcare providers and patients.

## 2. Materials and Methods

**Figure 1:**
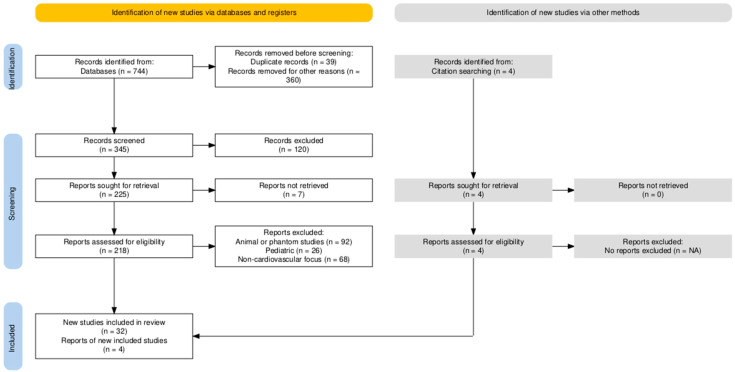
Literature review according to PRISMA guidelines.

This review was conducted to identify the strengths and limitations of needle navigation technologies for MICS that are currently deployed in clinical practice and to derive key requirements for an optimal needle navigation system. The review followed the Preferred Reporting Items on Systematic Reviews and Meta Analysis (PRISMA) guidelines [9]. The search protocol was not registered in advance. Systematic searches of the Web of Science, PubMed, and IEEE databases were conducted, focusing on needle navigation technologies used in MICS (Figure 1). Search queries included keywords and synonyms for “needle guidance” AND “cardiovascular” AND “minimal* invasive”. A total of 744 articles were identified from the start until 1 June 2024. Of these, duplicates and those published prior to 2014 or that do not focus on needle navigation technologies or methods were excluded, because this review focuses on the strengths and limitations of currently used technologies. The retrieval and data extraction process involved screening titles and abstracts for eligible criteria, followed by a full-text review to confirm eligibility. Eligibility criteria included original articles that performed needle navigation techniques in MICS in adult patients. Pediatric studies were not included, as procedures may differ significantly from those performed in adults due to the much smaller organs in children. Furthermore, case reports, clinical trials, and laboratory results were not included to avoid the risk of bias toward experimental or unique approaches and to focus only on methods that are currently actively used in clinical practice. However, these are certainly very valuable for novel technological approaches. After exclusions, 32 studies met the inclusion criteria and were included in the main review, supplemented by four relevant studies identified by the authors. All the reviewers verified the eligible articles independently.

### Data Extraction

Each study was reviewed by one author. Extracted data included the year of publication, number of patients, modalities used, procedures performed, and outcomes measured. If more than one modality or procedure was mentioned, only the modalities used for needle guidance and the procedure for which needle guidance was required were extracted. When two modalities were compared, the study was counted as a single-modality approach for one of the two modalities. Assessed outcomes included the advantages, limitations, strengths, and weaknesses of using the respective modality for needle navigation as mentioned by the article authors. From this, indications for further development, as demonstrated in actual patient use, were derived. Results from individual studies were presented in tabular form, categorized by key characteristics such as the type of modality used, overall technology used, modality 1 and, if available, modality 2. Outcomes were tracked and visualized with Microsoft Excel.

## 3. Results

### 3.1. Current State of Needle Navigation Technologies

This review analyzes the advantages and limitations of needle navigation technologies deployed in cardiovascular interventions over the past decade based on articles involving a minimum of five adult patients (Figure 1). A total of 744 articles were identified, of which 32 articles met the inclusion criteria, with 4 additional studies identified by the authors.

**Figure 2:**
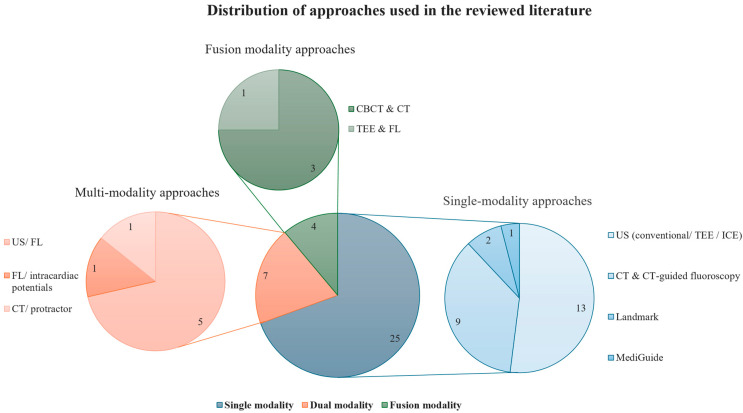
Distribution of approaches used in the reviewed literature. Single-modality approaches such as conventional ultrasound (US), fluoroscopy (FL), transesophageal echocardiography (TEE), intracardiac echography (ICE), or conventional computed tomography (CT) are used alone to guide the needle to the target site. Multi-modality approaches combine two single-modality approaches, while fusion modalities often utilize cone-beam CT (CBCT) images to overlay CT, TEE, or FL images.

Across those 36 studies, heterogeneity was observed in the technologies and modalities used, particularly between single-modality and multi-modality approaches (Figure 2).

The included studies were generally consistent in reporting the advantages and limitations of the respective techniques. The majority, comprising 25 studies, used single-modality approaches, such as standard US or CT-guided fluoroscopy. Seven studies used two of these single-modality approaches in parallel, resulting in multi-modality approaches. Four studies used fusion modalities combining multimodality images in one interface.

Studies deploying US include those using different probes, such as transesophageal echocardiography (TEE) or intracardiac echocardiography (ICE) (Table 1). Some studies compared different modalities, but they were only assigned to one modality [9,10,11].

### 3.2. The Landmark Technique

Historically, vessel punctures have been navigated via palpation using anatomical indicators, which is called the landmark technique. Despite its standardization, this method harbors risks, such as piercing through the posterior vessel wall, called posterior wall puncture (PWP). Furthermore, fingertip-guided palpation has a limited depth range of 2–4 mm, making it harder to discriminate deeper structures and difficult to use with more obese patients. Studies showed that US guidance significantly reduces the procedure complication rate—from 26% to just 6%—especially in patients with challenging access. US is particularly recommended for cases involving weak pulses from small or deep arteries, where palpation alone may be insufficient [10,13,29]. In such cases, an US device is either used routinely or as a rescue technique after several attempts to facilitate transradial access [29].

### 3.3. Needle Navigation with Ultrasound

#### 3.3.1. Conventional US

Of the 36 articles, 21 reported using US for needle visualization in cardiovascular procedures, with 10 studies deploying standard US probes and 11 utilizing either TEE or ICE probes alone or in conjunction with fluoroscopy. This made US the most common technique reviewed for MICS. US is highly effective in dynamic environments, such as the chest during breathing or in the vicinity of the beating heart, because it provides real-time visualization without ionizing radiation. It employs high-frequency sound waves transmitted into the body via a transducer probe, which are then reflected or scattered by anatomical structures to produce images. US is the recommended method for vessel access due to its real-time imaging and increased procedure precision. However, the penetration depth of standard US examinations is typically limited to 15 cm, with special probes up to 25 cm, posing challenges with obese patients. Due to its limited penetration depth, US is most effective for facilitating access to peripheral vessels in procedures like percutaneous coronary interventions (PCIs), coronary angiography, and central venous catheter placement (CVC) [5,6,7,10,13,14,15,20,24,29]. The orientation of metallic instruments, such as needles and catheters, significantly influences their visibility during US imaging because they can reflect the US beam and cause shadow and other artefacts [42]. Thus, choosing the access site is crucial for optimal visibility (Table 2) [9,13,29]. For instance, for accessing the heart in percutaneous coronary interventions (PCIs), the jugular vein is often preferred over the femoral vein due to the shorter paths and better outcomes involved, while for CVC, the distal radial approach (DRA) at the wrist is favored over the transradial approach (TRA) to avoid occlusion [9,15,43].

**Figure 3:**
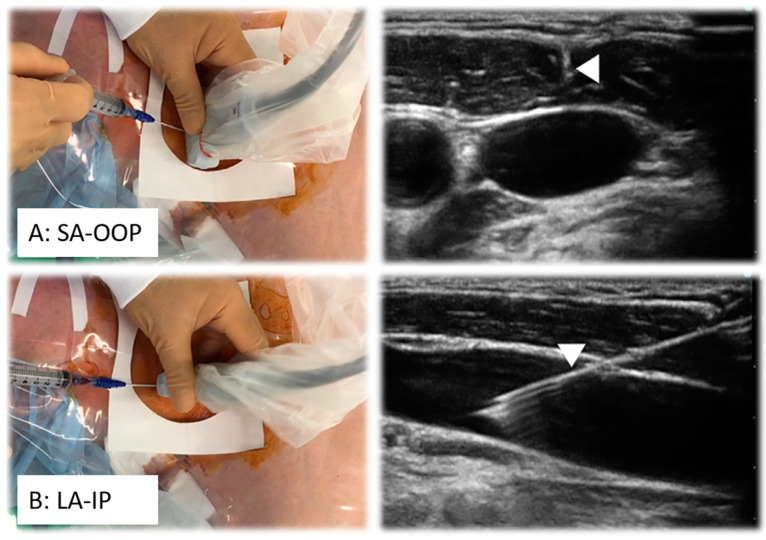
Schematic view of the long-axis in-plane (LA-IP) and short-axis out-of-plane (SA-OOP) approaches for needle (arrow) guidance. Adapted from [6] with permission from Elsevier.

Continuous needle visualization significantly reduces the number of punctures and the risk of complications by ensuring proper alignment of the US probe [12]. Two primary 2D single-plane methods guide this alignment: the short-axis out-of-plane (SA-OOP) approach and the long-axis in-plane (LA-IP) approach. The SA-OOP method displays the vessel as a circular cross-section with the needle perpendicular to the US beam, enhancing patient comfort due to a shorter needle path (Figure 3) [6,7]. On the downside, distinguishing the needle tip from the shaft becomes more challenging as the needle advances and increases the risk of PWP of adjacent vessels. In the LA-IP approach, the vessel appears in a longitudinal section as a truncated tube. The needle is seen as one white line, making it easier to follow, as in SA-OOP. In the LA-IP view, the needle has a longer path to travel and, therefore, causes more discomfort to the patient.

Less frequently combined approaches like combined short-axis and long-axis (CSLA) or dynamic needle-tip positioning (DNTP) are used to leverage the strengths of SA-OOP and LA-IP while mitigating their downsides. Both approaches benefit from the strengths of SA-OOP and LA-IP while disregarding the downsides [5,6,7]. More advanced techniques, such as 3D US, fuse SA-OOP and LA-IP images simultaneously into 3D ultrasound images and enhance the amount of information available during procedures [17].

Transthoracic echocardiography (TTE) provides direct visualization of the heart and surrounding vessels, with the US probe placed between two ribs [12,16,32]. Therefore, the space between two ribs limits the window size for the target site and needle visualization.

Although US is technically easy to manipulate, the actual application of this imaging modality is particularly difficult because it requires the continuous and simultaneous movement of both hands [6,44]. In addition, orientation to the target site and interpretation of the needle angle while manipulating the needle-probe alignment in parallel is challenging. Overall, US provides good soft-tissue contrast but requires an experienced operator to interpret and manipulate the images provided.

#### 3.3.2. TEE and ICE

For procedures that require direct access to the heart and, therefore, deeper visualization, such as for transseptal punctures (TSPs) or valve repair, TEE and ICE are the preferred navigation techniques [19]. TEE involves advancing a small US probe into the esophagus, enabling clear visualization of the adjacent heart [9,11,34]. However, standard TEE only offers 2D images, making navigation challenging. The development of 3D TEE has enhanced the visualization of cardiac anatomy and interventional tools, addressing previous limitations in 2D imaging [9,33]. A TEE and 3D TEE probe can also be used as a standard vascular surface probe and, as such, provide the same information for vascular access [18]. However, TEE and 3D TEE carry the risk of aspiration when performed in the supine position and may require intubation or anesthesia for longer procedures [37].

This downside is overcome by the ICE method, which uses small-sized, low-frequency, multi-directional steerable intracardiac catheters with built-in US probes, offering superior 2D visualization without the need for anesthesia [19]. ICE can be used intracardially or intravenously and provides direct imaging of the puncture site and related anatomical structures. It has also been shown to reduce procedural times. However, it is limited by its small monoplane image section and high cost due to the single-use nature of the catheters [19]. Despite these limitations, ICE is preferred over TEE/3D TEE for verifying device positioning and observing the intracardiac region during TSPs [37]. Nevertheless, both TEE/3D TEE and ICE require special training, which increases the overall procedural costs [33,37].

### 3.4. Needle Navigation with CT Guidance

Computed tomography (CT) and CT-guided fluoroscopy represent the second most common imaging techniques for needle guidance, with nine articles utilizing them as single-modality technologies and eleven incorporating them into multi-modality or fusion approaches. In CT, an X-ray source moves rotationally around the patient, clearly visualizing dense structures (e.g., bones, medical devices) but being limited in soft-tissue contrast, which can be improved with contrast agents. On the downside, the bundled X-ray beams expose both the patient and practitioner to radiation.

Of the reviewed studies, only two employed conventional CT imaging for needle guidance. One study used CT to plan the epicardial access [20]. Another study used preprocedural CT scans in conjunction with a protractor to successfully guide an MICS [31]. Twelve studies used fluoroscopy either alone or in conjunction with US techniques. Conventional CT provides static, high-resolution 3D images of tools and dense anatomical structures with a wider field of view than US. CT examinations create multiple image sets, which require some post-processing time to display CT images and, therefore, lack real-time imaging [24]. In addition, the devices must also be readjusted for each image set, increasing radiation exposure to patients and practitioners [24,39]. Therefore, CT fluoroscopy, which offers real-time imaging, albeit mostly in 2D depending on the method, is preferred for procedures in the dynamic cardiovascular environment [21,22,23,24,25,26,27,28,37]. Compared with US, fluoroscopy better visualizes deeper structures but lacks soft-tissue contrast and, often, 3D visualization [24]. Therefore, it is often combined with other modalities such as conventional US, TEE, or ICE [19,32,34,35,36,38]. Another option is the usage of rotational angiography that works on the same principle as fluoroscopy but focuses on the visualization of blood vessels and allows for a 3D reconstruction of the desired structures [23,33]. Therefore, it can help to augment fluoroscopy with 3D images [23,33].

Historically, TSP is performed under fluoroscopy alone or fluoroscopy in concordance with TEE [23,28,34,35,36,37]. Even when used in parallel, separate monitors for fluoroscopy and TEE challenge the radiologist to coordinate information of both modalities mentally [38]. Fusion technologies, such as Philips Healthcare’s EchoNav Release II tracking system, enable the real-time fusion of these images, reducing TSP procedure times [38]. Further improvements, like dynamic markers for systole/diastole or real-time 3D heart models, are required to improve orientation and the relevance of this technical development [38]. Other approaches for fusion modalities overlaid preprocedural CT angiograms or contrast-enhanced CT onto unenhanced intra-operative CBCT images to enable real-time multiplanar guidance [39,40]. This technique was used by Rhee et al. to guide translumbar type II endoleak treatment in hybrid rooms with needle trajectory and guidance software and allowed for precise real-time adjustments of the needle trajectory [39]. Using this approach, 26 patients were treated successfully with reduced contrast agent and radiation exposition [39]. In another study, contrast-enhanced CT-fluoroscopy fusion was used for transjugular intrahepatic portosystemic shunt placement and aortic aneurysm repair [40]. Based on a 3D virtual needle path that was overlaid onto 2D fluoroscopy for guidance, Tacher et al. were able to reduce the procedure time, fluoroscopy time, and radiation exposure as well as the use of contrast media [40,41].

### 3.5. Needle Navigation with MRI

None of the reviewed studies used MRI for needle guidance. Although MRI provides superior soft-tissue contrast, and real-time MRI (RT-MRI) exists for cardiovascular imaging, its incompatibility with metallic instruments limits RT-MRI’s application in needle navigation [8].

### 3.6. Requirements for an Optimal Needle Navigation System

In summary, each deployed modality in adult patients has its own strengths and limitations, making it currently difficult to achieve optimal guidance with a single technology (Table 3). Multimodal and fusion approaches that combine complementary techniques offer a promising strategy to compensate for individual weaknesses and to improve the overall navigation performance.

Examining the strengths and weaknesses of current needle navigation technologies highlights several critical features that an optimal needle guidance technology should incorporate to ensure precise, safe, and efficient MICSs (Table 4).Firstly, and most importantly, the technology must provide real-time data on the needle location within the body, guidance information toward the target site, and precise information on the proximity of the tip to this target site. This necessitates comprehensive spatial awareness of the dynamic 3D environment. Consequently, the technology should offer adequate soft-tissue contrast and device visibility at a centimeter scale, ensuring sufficient resolution for safe and accurate navigation. Moreover, real-time feedback on the structural integrity and condition of tissues traversed would provide clinicians with additional information to make flexible intraoperative adjustments. For versatile applications, the system should be reusable and support the combination of various needle types and standard tools without requiring proprietary or specialized equipment. This flexibility would also make the system cost-efficient and affordable to integrate into various existing clinical workflows without imposing substantial financial burdens. To maximize adoption, ease of use is equally important, and the technology should feature an intuitive interface requiring minimal training to allow seamless integration into established workflows.

## 4. Discussion

In MICS, needle guidance is crucial for procedural accuracy, patient safety, time efficiency, and success. Despite considerable research, an optimal solution for needle navigation that balances real-time orientation, the puncture site, and tool visualization, information about tissue integrity, compatibility with metallic tools, and affordability remains elusive.

Among the existing technologies, US and fluoroscopy are the most widely adopted single-modality approaches, largely due to their real-time imaging capabilities and device visibility. However, both modalities have notable limitations.

US represents the gold standard for peripheral vessel access due to its real-time imaging, portability, and cost-effectiveness, making it highly adaptable in dynamic environments. US equipment is widely available and cost-effective compared with CT or MRI, making it accessible to a broad range of healthcare facilities. This was supported by the fact that 58% of the analyzed literature used US for needle navigation. Its accessibility and flexibility is highlighted by studies optimizing needle navigation through simple probe adjustments [5,6,7]. However, US faces challenges in deeper tissue penetration, and the complicated manipulation required to coordinate the 2D alignment between US probes and instruments restricts its utility in complex cardiovascular procedures [20,24,44].

Three-dimensional US addresses the limitations of 2D navigation but still struggles with device visibility. By strategically manipulating the orientation of the US probe in relation to the needle, visibility can be enhanced, but this requires an experienced operator [17]. Furthermore, AI-driven automatic instrument detection software was shown to help needle navigation in 3D when compared with other existing state-of-the-art methods [44]. However, integrating this technology into existing US devices can pose a challenge. Another approach to enhance device visibility during 3D US examinations is to incorporate a fiber optic ultrasound sensor (FOUS) into the device’s tip or to use specialized sonographic needles [45,46,47]. The distance between the FOUS and the external imaging probe can be accurately measured through ultrasonic communication, thereby providing precise information about the tool’s localization [46,47,48]. Although ultrasonic needle tracking addresses the issue of device visibility, it introduces a new challenge: fiber optics are sensitive to bending, as can happen in cardiovascular catheterizations, such as when vessels abruptly bend [48]. Additionally, specialized equipment always limits technical flexibility and increases costs.

CT-guided fluoroscopy has become the gold standard for procedures requiring needle navigation and reliable visualization of deeper-located structures or the direct heart, where US often reaches its limit. Whereas conventional CT was only used in one study, 42% of the investigated studies used fluoroscopy either alone or jointly with other modalities. The lack of real-time imaging due to extended image processing times limits the broad adoption of conventional CT for cardiovascular needle interventions [24]. However, recent developments in artificial intelligence (AI) and AI-based mixed-reality (AR) techniques made it possible to augment static CT images virtually with previously planned needle trajectories in order to allow for real-time navigation [49,50,51]. The CT scan serves as the basis for 3D reconstructions of the patient’s anatomy. This virtual model is superimposed onto the patient’s body to navigate the needle according to the trajectory in real time using a head-mounted display, such as the HoloLens 2 [49,50]. The appeal of this technology lies in its potential to be applied to any imaging modality once it has been established. Furthermore, these 3D trajectories can also be integrated into robotic-assisted interventions, which have been shown to have similar accuracy to manual needle punctures in phantoms but with a significant reduction of the operator’s radiation exposure [52,53].

While CT-guided fluoroscopy already enables real-time imaging, its reliance on ionizing radiation and limited 2D visualization remain drawbacks. The MediGuide^TM^ released in 2012 partly overcomes this limitation by creating a 3D electromagnetic field that allows for the precise localization of medical devices. This spatial information is combined with preprocedural fluoroscopy images [30,54]. However, while the overall fluoroscopy time was significantly reduced, the high costs of the MediGuide system impedes its broader application [30]. This poses a common problem, as other advanced dual-modality systems also improve 3D visualization but often increase costs and operational complexity [19,32,34,35,36,38].

These findings emphasize that an optimal guidance technology should maintain real-time imaging and ease of use without imposing radiation exposure. They also highlight the need for sufficient tissue contrast and 3D visibility to support precise navigation within complex cardiovascular anatomies.

Although RT-MRI provides superior soft-tissue contrast in comparison with other imaging modalities, and the idea of using RT-MRI for cardiovascular procedure navigation was already present in 2009, it is not yet an established method for needle navigation due to its incompatibility with metallic instruments in the magnetic and high-frequency environment [8,55]. Instruments used within strong magnetic fields need to undergo extensive safety testing—magnetic attraction and heating—before they can be used (see ISO/TS 1010974 [56]). Most metallic medical devices are visualized passively in MRI, appearing as dark artifacts or negative contrasts. Therefore, RT-MRI requires custom-made needles, cannulas, and catheters made from shape memory alloy or glass-fiber epoxy-based materials to cause minimal interference with the outer magnetic field and incorporate small coils or antennae connected to the scanner to produce unique imaging signatures [57,58,59,60]. However, most research on MRI-compatible needles was conducted earlier than 2014, and although only some MRI-safe needles are commercially available, they have not been actively researched within the last decade [58,61,62]. Recent studies focused on the development and validation of those so-called “active” visible catheters in swine and in patients [36,57,59,60,63]. High acquisition costs for RT-MRI images and the limited availability of MRI-safe equipment limit the use of RT-MRI guidance in advanced centers and prevent its broader clinical adoption [8,55]. This reliance on proprietary, specialized, and often non-commercially available equipment not only elevates costs but also limits the entire system’s flexibility, thereby emphasizing the challenges a novel technology has to face.

Given that even US, the most used modality, only meets 60% of the requirements for optimal needle technologies, it seems reasonable that recent deployments and research have shifted toward hybrid approaches that combine multiple imaging modalities to compensate for individual weaknesses. Commonly used are multimodal approaches, which use several imaging modalities in parallel, but these face challenges in workflow integration, as the coordination of information from both modalities is cognitively challenging [38]. Conversely, fusion modalities combine preprocedural 3D image data with real-time imaging data, such as fluoroscopy, to generate a single comprehensive fusion image. One common approach involves the combination of US and fluoroscopy, as seen in devices like the EchoNav, which aim to combine the 3D real-time imaging capabilities of TEE and the consistent visibility of needles offered by fluoroscopy [30,38,64]. Additionally, several image fusion approaches overlay preprocedural CT images onto intraprocedural fluoroscopy images. This adds 3D information to fluoroscopy and reduces procedural time and radiation exposure [39,40,41]. However, hybrid rooms, as used by Rhee et al., cost over 50% more per minute than conventional operation rooms (EUR 19.88 instead of EUR 9.45) [39,65]. It always needs to be evaluated if those high costs, which are mainly driven by construction and inventory costs, are outweighed by the health gain [65]. While these fusion technologies theoretically provide a comprehensive imaging solution, practical limitations—primarily the high costs and complexity of integrating such systems into existing clinical workflows—prevent their broad applications.

The current limitations in needle navigation may largely result from a predominant reliance on imaging modalities, possibly originating from the familiarity with visual guidance and the stringent technical requirements that new technologies must satisfy before their clinical adoption. Vision is widely acknowledged as the primary human sensory channel, followed by hearing and touch. However, MICS eliminates both direct visual cues and the tactile feedback previously provided by tool–tissue interactions in open surgeries. Consequently, this dependence on optimal visibility can become problematic, as even advanced imaging may not always deliver sufficient visibility. Expanding sensory feedback through additional channels—such as restoring the haptic input and enhancing the auditory feedback—presents a logical and promising approach to enhancing the efficacy and safety of cardiovascular procedures.

Emerging technologies that enhance sensory feedback in MICS show promise in supplementing or returning lost haptic cues. Recent studies have demonstrated that acoustic signals derived from tool–tissue interactions allow various synthetic materials to be distinguished, indicating unique acoustic signatures among tissues [66,67,68]. Those vibroacoustic signals allow for real-time tissue event detection, which holds information about tissue structure and integrity, aiding in the avoidance of critical anatomies and enhancing procedural safety [67]. Additionally, they offer the potential for automated tissue classification, giving surgeons an alternative sensory channel [68,69].

Other approaches explore optical and photoacoustic methods for tissue characterization. For example, needles with embedded optical fibers use light transmission to detect tissue density and scattering properties, differentiating between healthy and pathological tissues within 1–2 mm of the needle tip [70]. Additionally, laser integration in ultrasound probes enables photoacoustic sensing to measure tissue characteristics in real time, similar to optical needles but without the need for specialized needles [71,72]. In a third approach, a novel mechanical injector provides accurate fluid delivery to the target site by sensing changes in tissue resistance [73]. While this device enhances targeting accuracy, it lacks modularity and requires specially modified syringes, which limits its versatility and increases the complexity in its clinical use [73]. Together, these technologies highlight varied approaches to enriching the sensory input and complement existing modalities, although each faces unique limitations and needs further research before its clinical adoption.

This review was naturally limited by the inclusion criterium requiring studies to have more than five adult patients, which excluded clinical trials and case reports that often showcase experimental and novel techniques. The focus, however, was to provide an overview of widely deployed methods. Additionally, techniques used in adults may differ from those in pediatric populations, warranting a separate analysis for pediatric applications. Furthermore, laboratory trials were excluded, as while analyzing currently investigated techniques in experimental settings could provide valuable insights, this was beyond the scope of this review.

## 5. Conclusions

Current needle navigation technologies predominantly rely on imaging methods, indicating that alternative techniques have yet to provide a comparable substitute for visual guidance. This may stem from the familiarity of visual cues in guiding procedures, as well as the stringent regulatory requirements that newer technologies must meet for clinical application. Thus, exploring innovative solutions to expand upon vision with sensory feedback during these procedures is a logical progression to improve outcomes. By extracting the criteria and challenges that an ideal needle navigation system must address prior to clinical adoption, this review aimed to facilitate the development of new technologies and to encourage the proposal of novel approaches.

To address these challenges, an ideal approach could involve integrating complementary sensory feedback channels, such as vibroacoustic or optical technologies, with existing imaging modalities. By complementing current systems rather than replacing them, new technologies can enhance the surgical toolkit and enable surgeons to gain additional information while preserving familiar workflows. Successfully fusing the information derived into a cohesive and intuitive system for needle navigation in MICS may ultimately enhance procedural safety, precision, and the overall surgical experience.

## Figures and Tables

**Table 1:** Overview of the assessed studies, categorized by the technology and modalities used, with their corresponding references.

Dominant Technology		Modality 1	Modality 2	References
Single-Modality	Ultrasound (US)	Conventional US	-	[5,6,7,12,13,14,15,16,17]
		TEE	-	[9,18]
		ICE	-	[11,19]
	Computed tomography (CT)	Conventional CT	-	[20]
		CT-guided fluoroscopy	-	[21,22,23,24,25,26,27,28]
	Landmark	Palpation	-	[10,29]
	MediGuide^TM^	-	-	[30]
Multi-Modality	CT	Conventional CT	Protractor	[31]
		CT-guided fluoroscopy	Conventional US	[32]
			TEE	[33,34,35,36]
			Intracardiac potentials	[37]
Fusion-Modality	US	TEE	CT-guided fluoroscopy	[38]
	CT	Conventional CT	Cone-beam CT (CBCT)	[39,40,41]

**Table 2:** Popular targets visualized with ultrasound imaging and their access sites.

Imaging Modality	Access	Target Site
**Conventional ultrasound**	Probe is aligned above the peripheral target site	Femoral or jugular vein for central venous catheterization
**Transthoracic echocardiography (TTE)**	Probe is aligned at the thorax between two ribs	Direct heart visualization
**Transesophageal echocardiography (TEE)**	Probe is advanced through the mouth into the esophagus	Direct heart visualization
**Intracardiac echocardiography (ICE)**	Probe is advanced through a vessel directly into the heart Probe is advanced into a vessel	Internal heart anatomy e.g., for transseptal puncture Internal vessel anatomy
**3D Transesophageal echocardiography (3D TEE)**	Probe is advanced through the mouth into the esophagus	Enhanced cardiac anatomy visualization with improved interventional guidance

**Table 3:** Strengths and weaknesses of currently deployed needle navigation techniques in minimally invasive cardiovascular surgeries.

Modality	Strengths	Weaknesses
**Landmark Technique**	Simple, always accessible, fast	Limited depth range (2–4 mm), higher risk of posterior wall puncture (PWP), not suitable for deeper or complex cases
**Ultrasound (US)**	Real-time imaging, widely adapted, non-radiation, versatile for peripheral vessel access, 2D and 3D options available	Limited penetration depth (25 cm max), instrument visibility depends on access site and orientation, high experience needed
**Transesophageal Echocardiography (TEE)**	Clear heart visualization, suitable for deeper procedures, 3D TEE offers improved anatomical visualization	Risk of aspiration, may require anesthesia or intubation, high experience needed
**Intracardiac Echocardiography (ICE)**	Direct imaging of puncture site, avoids anesthesia, reduces procedure time	Small monoplane image section, high costs due to single-use catheters, high experience needed
**Computed Tomography (CT)**	High-resolution, 3D visualization, large field of view	Radiation exposure, lacks real-time capability without fluoroscopy, limited soft tissue contrast, requires contrast agents for some applications
**Fluoroscopy**	Better visualization of deeper structures than conventional CT, real-time imaging, 2D and 3D options available, flexibility	Radiation exposure, limited soft tissue contrast
**Fusion Technologies**	Combines multiple modalities (e.g., EchoNav, CBCT & fluoroscopy) for enhanced visualization, reduces procedure time and radiation	Complex setup and integration in existing workflows, often very expensive (e.g., hybrid rooms)
**Real-time Magnetic Resonance Imaging (RT-MRI)**	Excellent soft-tissue contrast, potential for real-time cardiovascular imaging	Incompatibility with metallic instruments, not standardized for current needle navigation, very high costs

**Table 4:** Requirements for an optimal needle navigation technique compared with current technologies.

Requirement	Optimal Needle Navigation Technology	US	Fluoroscopy	RT-MRI
**Real-time information about needle localization**	Yes	Yes	Yes	Yes
**Target site localization**	Excellent, full visualization of the target site	Excellent, especially with ICE and TEE	Moderate, limited soft tissue contrast	Excellent (high-resolution)
**Needle visualization**	Excellent, full visualization of the needle	Moderate	Excellent	Limited (requires special MRI-compatible needles)
**Radiation**	No	No	Yes	No
**Soft tissue contrast**	Excellent	Good (variable based on settings)	Poor, soft tissue differentiation is difficult, contrast agents needed	Excellent
**Real-time feedback on the condition and integrity of tissues**	Yes	No	No	No
**Possible versatile application**	High	High, used in various cardiovascular procedures	Moderate, more specific applications	Low, limited by compatibility
**Integration in existing clinical workflow**	High	High, commonly integrated into routine procedures	Moderate, often used alongside other imaging modalities	Low, requires specialized settings
**System costs**	Low	Low	Moderate	High
**Training required**	Low	High	High	High
**Met Requirements**	**10**	**6**	**2**	**4**

## Data Availability

Data is available from the lead contact Katharina Steeg (katharina.steeg@radiol.med.uni-giessen.de).
